# Parents’ Knowledge On, Attitude Toward, and Practice of Screen Time Exposure Regulation of Their Children Under Six Years of Age in Western Region, Saudi Arabia

**DOI:** 10.7759/cureus.49464

**Published:** 2023-11-26

**Authors:** Safa H Alkalash, Faisal A Alshamrani, Saleh A Alharthi, Muslih A Alzubaidi, Rahaf M Alqarehi, Abdurahman A Bazaid, Bushra Asiri

**Affiliations:** 1 Community Medicine and Health Care, Umm Al-Qura University, Al-Qunfudah, SAU; 2 Family Medicine, Menoufia University, Shebin Elkom, EGY; 3 Medicine, Al-Qunfudah College of Medicine, Umm Al-Qura University, Al-Qunfudah, SAU; 4 College of Medicine, Umm Al-Qura University, Makkah, SAU; 5 College of Medicine, King Saud Bin Abdulaziz University for Health Sciences, Jeddah, SAU; 6 College of Medicine, Ibn Sina National for Medical Sciences, Jeddah, Saudi Arabia, Jeddah, SAU

**Keywords:** parents, computers, televisions, screen time, children

## Abstract

Background: Children and teenagers spend a significant amount of time watching screens, which include cellphones, tablets, gaming consoles, televisions, and computers. Regulation of screen time exposure is a crucial matter to avoid the health drawbacks of prolonged screen exposure.

Objectives: Assessment of parents’ knowledge, attitude, and practice of regulation screen exposure among their children under six years old in the western region of Saudi Arabia.

Methods: A cross-sectional study was conducted on a convenience sample of 451 parents of under-six-year-old children in the western region of Saudi Arabia. Data were collected by using an online questionnaire, and a link to the survey was distributed to respondents via electronic platforms as well as to primary healthcare visitors. The data were analyzed using SPSS software.

Results: This study included 451 parents from the Saudi western region. Most of the participating parents were female (64.3%), aged 25-34 years (34.8%), married (86.0%), and had completed their university education (56.1%). This study found that 76.4% of parents had adequate knowledge, 73.1% had a positive attitude, and 69.8% had adequate practice of screen time regulation for their children under six years of age. Factors associated with their good knowledge include being married (p = 0.002), having government work (p = 0.020), having children who use mobile phones, and having children attend kindergarten (p <0.001) for each. Furthermore, highly educated parents showed more positive attitudes than others (p-value = 0.004). Finally, better practice of screen time regulation was noted among highly educated parents (p = 0.011), who had government jobs (p = 0.031), and children who went to kindergarten (p = 0.031) for their children.

Conclusion: In this study, parents of under-six-year-old children possessed overall good knowledge and a positive attitude, but their actual practice of screen time regulation for their children was low, specifically in terms of parental supervision of the content that children watch, their care of children’s regular exercise, and using devices as a method for motivating or punishing their children. Additionally, sociodemographic characteristics like education, occupation, and marital status played a role in this issue. Healthcare professionals such as pediatricians, family doctors, general practitioners, and others can caution parents of young children under six years old about the harmful consequences of excessive screen use. Further longitudinal research is needed to understand the long-term effects of screen time exposure among young children. From this study, further qualitative research would aid in a greater understanding of the impact of parental knowledge, attitude, and practice on their children’s use of screen time.

## Introduction

Screen time refers to the amount of time an individual spends using electronic devices that have screens, such as televisions, computers, tablets, game consoles, and smartphones [1]. Over the past two decades, mobile phones and computers have grown, increasing our reliance on these technologies in our daily work [2]. Children are one of the most avid consumers of technology [3].

Good habits, such as physical exercise and getting enough sleep, are considered some of the leading factors associated with better health and psychosocial status in children and youth [4-6]. Previous studies have found associations between screen time and less physical activity, mental health, and shorter sleep duration [7]. The use of technology such as television, laptops, smartphones, and tablets can negatively affect the health of children, especially by increasing illness and reducing physical activity. Children who excessively use technology are more likely to develop obesity, type 2 diabetes, and miscellaneous medical and psychological problems [8,9]. In prekindergarten children, there is a link between greater screen-based media use and decreased microstructural integrity of brain white matter tracts supporting language and emergent literacy skills [10]. Additionally, a study found that the longer the time spent on screen time, the greater the child’s behavior problem [11]. In 2019, an article highlighted the idea that the home environment is highly associated with factors contributing to physical inactivity and increasing screen time and its association with overweight and obesity in children, and parents are concerned even as they generally show a lack of confidence in controlling their children [12].

The American Academy of Pediatrics recommends considering the quality of interactions with digital media and not just the quantity or amount of time. It’s crucial to consider the specific online activities that young children and teenagers partake in and to encourage them to use social media in ways that build their social, emotional, cognitive, and identity development [13]. Additionally, the American Academy of Child and Adolescent Psychiatry states that managing a child’s screen time is challenging for families and puts out guidelines to help parents manage this issue, like: until 18 months of age, limiting screen use to video chatting with an adult (for example, with a parent who is out of town); between 18 and 24 months, screen time should be limited to watching educational programming with a caregiver; and limit non-educational screen time to about one hour per weekday and three hours on the weekend days for those ages two to five [14]. Children ages six and older should be encouraged to develop healthy habits and limit activities that include screens [14]. During family meals and trips, all screens should be turned off. Avoid using screens as pacifiers, babysitters, or to stop tantrums; turn off screens and remove them from bedrooms 30-60 minutes before bedtime [14].

Mobile devices are unquestionably appealing since they are convenient to use and carry. Additionally, they assist parents in keeping their young children occupied when they need to finish activities and take a break from a very active day. They have inexpensive access to a wide variety of leisure and informational resources [15]. For better or worse, technology has a growing impact on adults’ lives, and young children are becoming a part of this reality. Young children can learn to read and speak by playing with or using interactive media that has been properly developed for them, but they still need adult supervision and contact [16,17]. There are no previous studies that evaluated parents’ knowledge, attitude and practice with their under-six-year-old children about screen time exposure in the western region of Saudi Arabia. Thus, this study was executed to highlight this important issue.

## Materials and methods

Study design

A descriptive cross-sectional study was carried out to assess parents’ knowledge of, attitude toward, and practice of regulation screen exposure among their less than six-year-old children in the western region of Saudi Arabia from April 2023 to August 2023.

Study setting

The research study was carried out in the western part of Saudi Arabia, which is divided into 26 governorates between its two main provinces, Makkah (17 governorates) and Al-Madinah (9 governorates). The responses were obtained from parents who were living in Makkah, Madinah, Jeddah, Al-Qunfudah, Al-Taif, and Al-Ardiyat.

Sample size estimation

The sample size was calculated using EPI-info (Centers for Disease Control and Prevention, Atlanta, Georgia) based on a total population in the Saudi western region of 8,325,304, with a confidence interval of 95% and a 5% margin of error. The minimum sample size was 384.

Tool and procedure for data collection

The study’s authors selected the survey questions after thoroughly looking over the literature and consulting the research team [12-15]. Finally, a consensus was obtained from all members of this team about the items that reflect the research question. The survey contents were subdivided into four sections: the first section was about socioeconomic data, such as age, gender, marital status, education level, employment, and monthly income, as well as questions about the lifestyle of the children, like quality of sleep and their physical activity. The second section involved 20 questions (some of them were multiple choices while the others were true or false) to assess the parents’ practice for regulating their children’s screen time and focused on the type of device used, the number of hours spent on the device per day, and the type of content the child is watching on the device. The third section involved six questions (3-point Likert scale) that were asked to find out the parents’ attitude toward their children’s use of electronic devices, such as using their devices under the parent’s supervision, and their feelings of concern about the children’s use of these devices. Finally, eight multiple-choice questions evaluated the parents’ knowledge of recommended screen time and the negative effects on the quality of sleep, physical activity, and communication skills with friends or parents.

A pilot study of 26 participants was undertaken, and the questionnaire was pre-tested to check language clarity and question understandability. Considering the instrument’s reliability, it was analyzed with a Cronbach’s alpha coefficient of 0.89. It gave us an idea of the time needed to answer the survey questions, but there was no need to modify any of the survey’s components.

Data were collected by using an online questionnaire created using a Google Form application, and its electronic link was distributed to respondents via WhatsApp groups and telegram groups of parents in schools, as well as to visitors to primary healthcare centers, to assess the effect of screen time and video gaming on their children. The survey had an opening question that assessed whether the participant was from the western region or not and if he or she could participate in it; otherwise, it was not allowable.

Scoring of knowledge, attitude, and practice

For the eight knowledge questions, the following common scoring system was employed: Correct responses received 1 point, while unresponsive and incorrect ones received 0. Participants were deemed to have low knowledge as they received fewer than 60% of the correct answers, while high knowledge was decided when they received at least 60%. There were two categories for rating their attitudes: agree (1 point) and disagree or neutral (0 points). A participant was deemed to have a positive attitude at a score of 60% or more; otherwise, they were considered to have a negative attitude. The same scores were used to differentiate between good practice (60% or more) and poor practice (less than 60%) [16].

Ethical considerations

Ethical approval for the study was obtained from the research ethics committee of Umm Al-Qura University, Approval number: (HAPO-02-K-012-2023-05-1612). In order to obtain informed consent from each participant and guarantee the confidentiality of the data obtained, an introductory question was included in the questionnaire. All information was carefully labeled and handled to ensure its security, and no participant-specific personal information was collected.

Data analysis

The data were statistically analyzed using the SPSS application version 26. The research data were qualitative and expressed in numbers and percentages. To assess the relationship between the variables, the Chi-squared test (χ2) was applied to qualitative data, and the Fisher’s exact test was applied to distributions with small frequencies. Statistical significance was set up as a p-value of 0.05.

## Results

Over a period of five months, 477 replies were submitted in total. Twenty-six surveys were invalid or incomplete; therefore, they were discarded. Consequently, there were 451 final, valid, and complete questionnaires. Most of them were female (64.3%, n = 290), aged between 25 and 34 years (34.8%, n = 157), and half of them were from Makkah and its preservations (51%, n = 231). The majority were married (86.0%, n = 338) and had completed their university education (56.1%, n = 253). About a quarter had a monthly income of 10000-20000 SAR (2700-5400 $), which represented 27.1% (n = 122), and government jobs were the most common profession (40.6%, n = 183). The number of children under the age of seven varied, with 78.3% (n = 353) having less than three children, mostly 57.4% of the boys (n = 259). Additionally, 57.6% (n = 260) of children attended kindergarten (Table 1).

**Table 1:** Sociodemographic characters of parents and their children

		Frequency (n=451)	Percent
Gender of parents	Female	290	64.3
	Male	161	35.7
Age in years	15-24	72	16.0
	25-34	157	34.8
	35-44	141	31.3
	More than 45	81	18.0
Nationality	Saudi‎	451	100.0
Residence	Makkah and its preservations	231	51.0
	Medina and its provinces	220	48.6
Social status of parents	Husband/Wife	388	86.0
	Separated/Divorced	40	8.9
	Widowed	23	5.1
Academic qualifications	Primary	28	6.2
	Intermediate	12	2.7
	Secondary	63	14.0
	Diploma	39	8.6
	University	253	56.1
	Postgraduate	56	12.4
Monthly income	Less than 5000 SAR	104	23.1
	5000-10000 SAR	124	27.5
	10000-20000 SAR	140	31.0
	More than 20000 SAR	83	18.4
Professions	Government job	183	40.6
	Housewife	114	25.3
	Private job	69	15.3
	Student	47	10.4
	Independent business	38	8.4
Number of children under the age of six years	Less than three years	353	78.3
	Equal or more than three years	98	21.7
Child gender	Male	259	57.4
	Female	192	42.6
Child goes to kindergarten	Yes	260	57.6
	No	191	42.3

The older children (three years and older) had a higher proportion of children who possess their own electronic device (56.6%) compared to the younger group (26.3%) (p <0.001). The older children (three years and older) had a higher proportion of children using tablets (like iPads) (45.3%) compared to the younger group (38.1%), but this difference was not statistically significant (p = 0.460). Older children spent 1-2 hours on electronic devices, increasing their time using devices compared to those less than three years old (p-values = 0.026, 0.032, respectively), while parents of younger children allow them to use devices only beside their parents and engage them in physical activities compared to parents of older children (p-values =0.034, 0.017, respectively) (Table 2).

**Table 2:** Under-six-year children’s total screen time spent at home, comparison between two age groups *Fisher’s exact test; p-value less than 0.05 is significant.

		Child age in years		p-value
		<3 years	3-6 years	
		N=186 (%)	N=265 (%)	
Your child has his own device.	No	137(73.7)	115(43.4)	<0.001
	Yes	49(26.3)	150(56.6)	
Type of device used by a child	iPad	48(38.1)	102(45.3)	0.460*
	Smart Phone	59(47.6)	86(38.6)	
	PlayStation/Xbox	16(12.6)	32(14.2)	
	Computer/Laptop	2(1.5)	4(1.7)	
Time allowed by the child to spend on screen daily	< 1 hour	67(36.0)	70(26.4)	0.026
	1-2 hours	52(28.0)	106(40.0)	
	2-3 hours	13(7.0)	24(9.1)	
	More than 3 hours	54(29.0)	65(24.5)	
Device use increased after the COVID-19 pandemic	No	56(30.1)	63(23.8)	0.032
	Yes	82(44.1)	150(56.6)	
	Maybe	48(25.8)	52(19.6)	
Allow son to use devices near you	No	13(7.0)	15(5.7)	0.034
	Yes	129(69.4)	157(59.2)	
	Maybe	44(23.7)	93(35.1)	
You set a time limit for your child to use the device or watch TV.	No	53(28.1)	86(32.5)	0.370
	Yes	133(71.5)	179(67.5)	
Your child accepts this limit without disobedience.	No	79(42.5)	116(43.8)	0.784
	Yes	107(57.5)	149(56.2)	
A child watches TV while eating a meal.	No	78(41.9)	104(39.2)	0.566
	Yes	108(58.1)	161(60.8)	
Your son suffers from obesity.	No	149(80.1)	209(78.9)	0.749
	Yes	37(19.9)	56(21.1)	
Your son engages in physical activity.	No	53(28.5)	54(20.4)	0.017
	Daily Basis	80(43.0)	103(38.9)	
	Weekly Basis	53(28.5)	108(40.8)	
You motivate children with smartphones or tablets as a reward.	No	52(28.0)	60(22.6)	0.367
	Yes	86 (46.2)	125(47.2)	
	Sometimes	48(25.8)	80(30.2)	
You forbid your child from using a device or watching TV before bedtime.	No	49(26.3)	54(20.4)	0.319
	Yes	98(52.7)	148(55.8)	
	Sometimes	39(21.0)	63(23.8)	
Your child sleeps early at night.	No	58(31.2)	87(32.8)	0.713
	Yes	128(68.8)	178(67.2)	
Your child uses a device or TV just before bedtime.	No	54(29.0)	76(28.7)	0.881
	Yes	86(46.2)	118(44.5)	
	Sometimes	46(24.7)	71(26.8)	
You motivate your child to participate in physical activities and play with his/her peers instead of staying on the devices.	No	18(9.7)	15(5.7)	0.107
	Yes	168(90.3)	250(94.3)	

**Figure 1:**
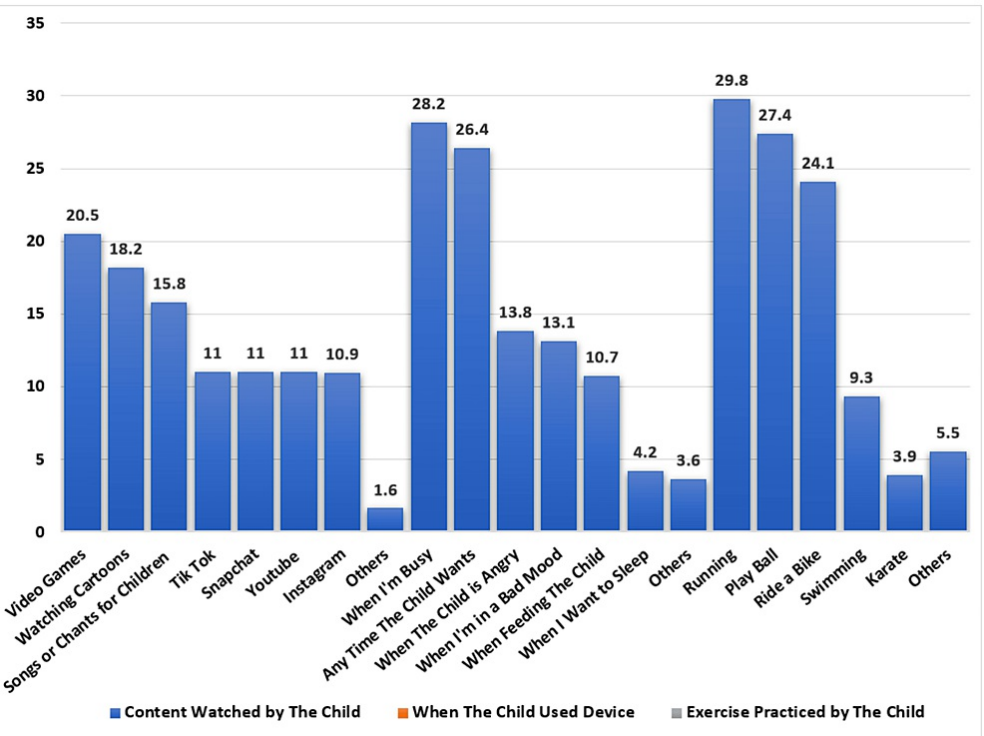
Content watched by children, time of screen exposure, and type of sports practiced by the children

Children most frequently watch cartoons (18.2%, n = 82), video games (20.5%, n = 85), and kid-friendly music or chants (15.8%, n = 71). When it comes to using electronics, 28.2% (n = 117) of kids are permitted to do so when their parents are at work, while 26.4% (n = 119) have unfettered access. Unexpectedly, 13.8% (n = 62) of kids who yell or act out are handed devices. A total of 344 (76.3%) children were incorporated into regular physical activity, whether daily or weekly. Running (29.8%, n = 103) and playing ball games (27.4%, n = 94) are the most popular sporting activities, followed by biking (24.1%, n = 83), then 9.3% (n = 32) of children participate in swimming, and 3.9% (n = 13) practice karate, while 5.5% (n = 19) of them practice other forms of exercise (Figure 1).

**Figure 2:**
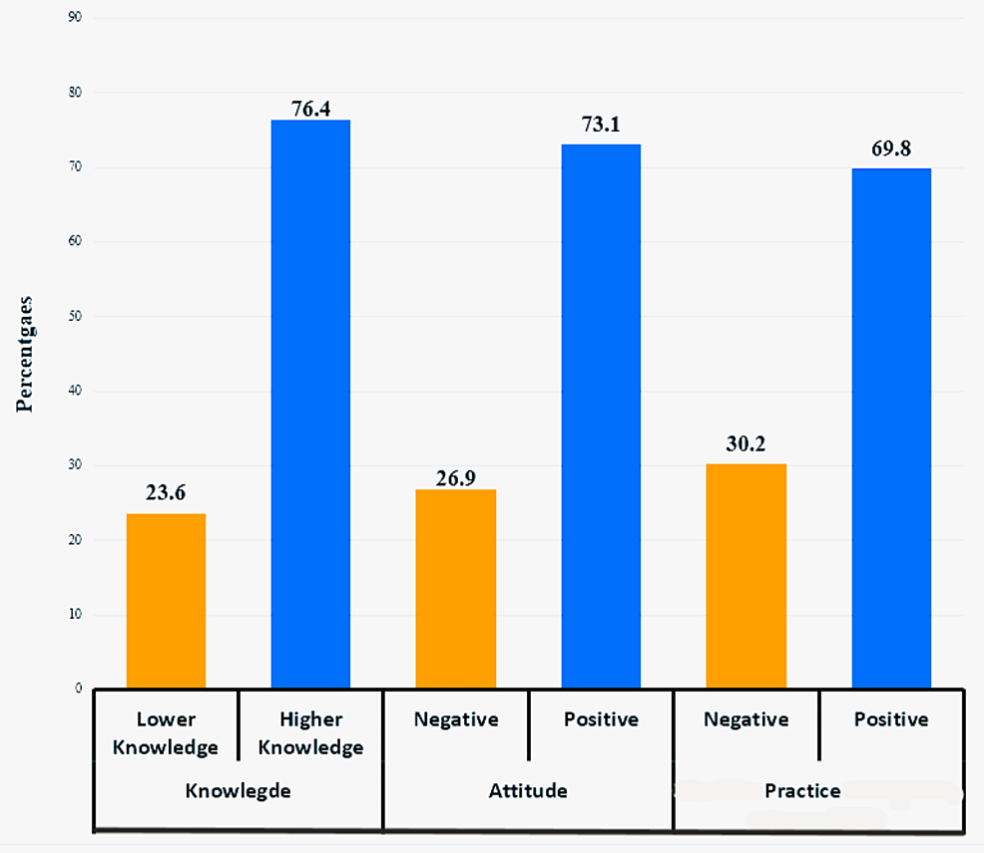
Levels of knowledge, attitude, and practice of parents toward regulation screen exposure

About 24% (n = 107) of parents have a lower level of knowledge, while 76.4% (n = 344) possess higher knowledge about the regulation of screen time exposure for their children. Concerning attitude, 26.9% (n = 121) exhibit negativity, while 73.1% (n = 330) show a positive attitude. In terms of regulating screen time, 30.2% (n = 136) of parents practice negatively, while 69.8% (n = 315) practice positively (Figure 2).

Gender showed a slight association, with better knowledge among females (71.9%, n = 213) than males (38.1%, n = 131) (p = 0.058). Age was not significantly associated with knowledge (p = 0.622). However, marital status strongly influenced knowledge, with a high proportion of husbands and wives (82.8%) having high knowledge compared to separated, divorced (10.8%, n = 37), or widowed (6.4%, n = 22) individuals (p = 0.002). Academic qualifications did not show any statistical significance (p = 0.089). Interestingly, parents with monthly income between 10000 and 20000 SAR (32%, n = 110) and those having government jobs (40.7%, n = 140) had higher knowledge than the others (p-values = 0.012, 0.020), respectively. Notably, the parents of the children who used a mobile phone tended to have higher knowledge (p < 0.001). Similarly, parents with children going to kindergarten demonstrated higher knowledge (66% vs. 34% without) (p < 0.001) (Table 3).

**Table 3:** Factors associated with the level of knowledge of parents regarding screen time of their child * Fisher's exact test; p-value less than 0.05 is significant.

		Level of knowledge		P-Value
		Poor (N=107)	Good (N=344)	
Gender	Female	77	213	0.058
	Male	30	131	
Age	15-24 Years	21	51	0.622
	25-34 Years	38	119	
	35-44 Years	31	110	
	More than 45 Years	17	64	
Residency	Makkah and its preservations	52	179	0.535
	Medina and its provinces	55	165	
Marital status	Husband/Wife	103	285	0.002*
	Separated/Divorced	3	37	
	Widow	1	22	
Academic qualifications	Primary	5	23	0.089*
	Intermediate	0	12	
	Secondary	11	52	
	Diploma	12	27	
	University	69	184	
	Postgraduate	10	46	
Income	Less than 5000 SAR	32	72	0.012
	5000-10000 SAR	35	89	
	10000-20000 SAR	30	110	
	More than 20000 SAR	10	73	
Occupations	Government job	43	140	0.020
	Housewife	35	79	
	Private job	9	60	
	Student	15	32	
	Independent business	5	33	
Child uses mobile phone	Yes	20	209	<0.001
	No	87	135	
Child goes to kindergarten	Yes	33	227	<0.001
	No	74	117	

Overall, most parents demonstrated a positive attitude toward screen time regulation for their children less than six years old (73.2%, n = 330), irrespective of their gender, age, residency, income, occupation, child’s mobile phone use, or kindergarten attendance. However, there were significant relations between parents’ academic qualifications (p = 0.004) and their attitude toward screen time regulation of their children, as parents with higher education, like universities (55.8%, n = 184), showed a more positive attitude (Table 4).

**Table 4:** Factors associated with the attitude of parents toward the incidence of screen time of their child *Fisher's exact test; p-value less than 0.05 is significant.

		Attitude of parents		P-Value
		Negative (N=121)	Positive (N=330)	
Gender	Female	81	209	0.431
	Male	40	121	
Age	15-24 Years	22	50	0.601
	25-34 Years	46	111	
	35-44 Years	35	108	
	>45 Years	18	61	
Residency	Makkah and its preservations	61	169	0.678
	Medina and its provinces	60	161	
Marital status	Husband/Wife	105	283	0.846
	Separated/Divorced	11	29	
	Widow	5	18	
Academic qualifications	Primary	1	27	0.004*
	Intermediate	1	11	
	Secondary	20	43	
	Diploma	12	27	
	University	69	184	
	Postgraduate	18	38	
Income	Less than 5000 SAR	27	77	0.975
	5000-10000 SAR	32	92	
	10000-20000 SAR	39	101	
	More than 20000 SAR	23	60	
Occupations	Government job	43	140	0.649
	Housewife	31	83	
	Private job	20	49	
	Student	14	33	
	Independent business	13	25	
Child uses mobile phone	Yes	64	167	0.667
	No	57	163	
Child goes to kindergarten	Yes	67	193	0.553
	No	54	137	

Regarding gender, there was no significant difference in the practice of screen time regulation between female and male parents. Age also did not show a significant association with screen time regulation. Similarly, residency and marital status did not appear to be strongly correlated with the practice. However, academic qualifications did show some significance, with parents with a university education or higher being more likely to have a positive practice of screen time regulation (p = 0.011). Occupation also played a role, as parents with government jobs were more likely to have positive practices (p = 0.020). Interestingly, the number of children under seven did not significantly influence the screen time regulation practice. However, if the child used a mobile phone or attended kindergarten, it was associated with a more positive practice among parents (p = 0.031) (Table 5).

**Table 5:** Factors associated with the practice of regulating the screen time of their children *Fisher’s exact test; p-value less than 0.05 is significant.

		Practice of parents		P-value
		Poor (N=136)	Good(N=315)	
Gender	Female	90	200	0.585
	Male	46	115	
Age	15-24 Years	25	47	0.139
	25-34 Years	54	103	
	35-44 Years	40	101	
	More than 45 Years	17	64	
Residency	Makkah and its preservations	65	166	0.339
	Medina and its provinces	71	149	
Marital status	Husband/Wife	122	266	0.274*
	Separated/Divorced	10	30	
	Widow	4	19	
Academic qualifications	Primary	3	25	0.011*
	Intermediate	3	9	
	Secondary	14	49	
	Diploma	6	33	
	University	92	161	
	Postgraduate	18	38	
Income	Less than 5000 SAR	24	80	0.065
	5000-10000 SAR	41	83	
	10000-20000 SAR	38	102	
	More than 20000 SAR	33	50	
Occupations	Government job	50	133	0.020
	Housewife	25	89	
	Private job	27	42	
	Student	17	30	
	Independent business	17	21	
Child uses mobile phone	Yes	62	167	0.148
	No	74	148	
Child goes to kindergarten	Yes	68	192	0.031
	No	68	123	

## Discussion

The present study aimed to assess parents’ knowledge, attitude, and practice regarding the regulation of screen exposure among their children under the age of six in Saudi Arabia’s western region. Various important findings in this study shed light on the parents’ and children’s sociodemographic characteristics, as well as factors associated with their knowledge, attitude, and practice regarding screen time regulation.

Many parents are concerned about screen time regulation for their young children. Most parents who participated were female, which is consistent with previous research showing that mothers often take a more active role in the childcare and supervision of young children [17]. Furthermore, a large proportion of parents were between the ages of 25 and 34, indicating that most of them were young parents. This age group is important for understanding screen time practices because they are more likely to be exposed to digital technologies and may have different perspectives on their children’s screen use than older generations [18].

More than half of children aged three to six years have a personal device, which is a questionable issue as this will increase the addiction to using such devices due to their availability. This is proved by the statistical significance that was found between both groups of children in terms of time spent on screen, where 40% of older children (3-6 years) spent one to two hours on their devices daily in comparison to 28% of the younger children (less than three years old). COVID-19 has an impact on electronic device use among children, as 56.6% of children aged three and older increased their device use after the COVID-19 pandemic. This may be due to the special context during the COVID-19 pandemic, like social distancing and the eruption of new online platforms at different levels of education and work. All make screen exposure a routine daily activity among all populations, including children. This was supported by many studies [19-21].

The age of the children and their screen time habits revealed some interesting patterns. When compared to children aged less than three years, children aged three to six years had significantly more screen time. This finding is consistent with previous research indicating that children’s screen time increases as they get older [22,23]. The three- to six-year-old age group also began using screens later and had higher screen permission, indicating a progressive trend of increasing screen exposure with age. This is concerning because excessive screen time during early childhood has been linked to negative developmental and health outcomes [24]. The study also discovered that a higher percentage of children aged less than three watched screens until they fell asleep, which could affect their sleep quality and overall well-being. This outcome could be considered a red flag to educate parents about the bad outcomes of allowing their children to watch screens during sleep time [25].

Children’s content consisted primarily of video games, cartoons, and children’s songs. These findings support previous research indicating that entertainment-related content is popular among young children [26]. Parents must be aware of the content their children are exposed to, as inappropriate or violent content can have a negative impact on their children’s behavior and emotional well-being [25]. Many parents permit electronic device use during children’s emotional outbursts. Though it may be suitable at times, this coping mechanism could unintentionally reinforce negative behavior, hindering the development of emotional regulation [27]. In terms of physical activity, the study found inadequate physical activity as a daily routine, specifically among older children (3-6 years) (p-value = 0.017). This is similar to a recent survey in Saudi Arabia, which indicates a worrying lack of knowledge and enthusiasm among parents about the right amounts of physical activity for their kids [28]. There is an urgent need for additional initiatives to counteract Saudis’ rising propensity for inactivity and unhealthy living.

Most parents had completed their university education. Higher education has been associated with a better understanding and awareness of child development and parenting practices [29]. This could be a contributing factor to the relatively good level of knowledge about screen time regulation (76.4%, n = 344) observed among the parents in this study. This finding is similar to that detected by another Saudi study in the Makkah region, which found that 77.3% of respondents were aware of the maximum amount of time children are permitted to use electronic devices, and according to their overall knowledge score, 78.7% had sufficient awareness about the amount of screen time children might have [22]. Moreover, the present study found that parents with government jobs exhibited higher levels of knowledge. This may be attributed to the fact that government jobs often require a certain level of education, and employees may have access to resources and training related to child development and health.

In this study, parents of children under six years old showed a positive attitude and good practice of controlling screen time exposure for their children, which is opposite to the findings of an Egyptian study [30]. The main cause of this difference may be related to the differences in both study settings and participant characteristics.

Factors like gender, marital status, education, occupation, a child’s mobile phone use, and kindergarten attendance influenced parents’ knowledge and practice of screen time exposure for their under-six-year-old children, highlighting the importance of education and environmental influences [31].

Strengths and limitations

This study’s strengths lie in the novelty of its topic within the Saudi context. Additionally, the inclusion of diverse sociodemographic backgrounds among parents makes the findings more representative of the region’s population. However, it is essential to acknowledge some limitations. Our study relied on self-reported data, which may be subject to recall bias or social desirability bias. Additionally, the cross-sectional design limited the ability to establish causality or assess changes in screen time practices over time. Therefore, qualitative research is recommended through organizing focus group discussions among parents to deeply recognize their perceptions about this important topic.

## Conclusions

There are some gaps in parents’ knowledge and practice regarding the supervision of their children while watching electronic devices, unlimited time of exposure, insufficient involvement of children in daily physical exercise, and using the device as a reward for their children, even though this study found adequate levels of parental knowledge, attitudes, and practices regarding screen time regulation for children under the age of six in Saudi Arabia’s western region. A high participation rate signifies parents’ concern for managing screen time. Education, occupation, and marital status influence parents’ awareness. Concerns arise from increased screen time with age and its potential negative effects, necessitating parental monitoring and alternative coping mechanisms. The study stresses the urgent need to organize educational campaigns for educating parents on responsible screen use and promoting physical activities, requiring collaboration among policymakers, healthcare professionals, and educators for children’s well-being in the digital age. Interventions to improve parental behavior may be an intriguing way to reduce preschoolers' overall screen time, but further research is required to validate this.

## Appendices

 **استبيان معرفة، ومواقف، وممارسات الإباء والامهات بشأن تنظيم تعرض أطفالهم تحت سن السادسة للشاشة في المنطقة الغربية، المملكة العربية السعودية**

هل ترغب بالمشاركة في الدراسة مع التأكيد من جانبنا على عدم نشر أي معلومات شخصية تخص المشاركين؟

نعم

لا

البيانات الديموغرافية  

1-مقر الاقامة؟

مكة المكرمة ومحافظتها

المدينة المنورة ومحافظتها

خارج المنطقة الغربية

2-العمر؟

3-الجنس؟                            ذكر              انثى

4-الجنسية؟                        سعودي           غير سعودي

5-الحالة الاجتماعية؟

زوج/ة

منفصل/مطلقة

أرمل/أرملة

6-المؤهل العلمي؟

ابتدائي

متوسط

ثانوي

دبلوم

جامعي

دراسات عليا

7-الدخل الشهري؟

اقل من ٥٠٠٠ ريال

٥٠٠٠ - ١٠٠٠٠ ريال

١٠٠٠٠-٢٠٠٠٠ ريال

اكثر من ٢٠٠٠٠ ريال

لا يوجد

8-المهنة؟

وظيفة حكومية

القطاع الخاص

ربة منزل

طالب

اعمال حرة

9-هل لديك اطفال تحت سن 6 سنوات؟

نعم                    لا

إذا كانت الإجابة ب “نعم"

10-ما هو جنس طفلك/ اطفالك؟        ذكر      انثى

11-كم عمر طفلك؟

12-هل طفلك يذهب لروضة الأطفال؟   نعم      لا

ممارسات الإباء والامهات بشأن تنظيم تعرض أطفالهم تحت سن السادسة للشاشة في المنطقة الغربية، المملكة العربية السعودية

1-هل لدى طفلك جهاز خاص به؟     نعم       لا

2-ما نوع الجهاز الذي يستخدمه؟

هاتف ذكي

ايباد

حاسوب/ لابتوب

بلايستيشن/ اكس بوكس

3-هل تسمح لأطفالك بمشاهدة التلفزيون أو استخدام الهواتف الذكية / الأجهزة اللوحية؟

نعم

لا

4-كم من الوقت يقضي أطفالك في اليوم على مشاهدة التلفزيون أو استخدام الهواتف الذكية / الأجهزة اللوحية؟

أقل من ساعة في اليوم

ساعة واحدة إلى ساعتين في اليوم

من ساعتين إلى ثلاث ساعات في اليوم

أكثر من ثلاث ساعات في اليوم

5-هل زادت ساعات استخدامه لجهازه بعد جائحة كورونا؟

نعم

لا

ربما

6-ما هو المحتوى الذي يشاهده طفلك؟

العاب فيديو

 يوتيوب

 تيك توك

 سناب شات

انستغرام

مشاهدة أفلام كرتونية

أغاني للأطفال

أخرى

7-هل تجعل ابنك يستخدم جهازه بالقرب منك؟

نعم

لا

أحيانا

8-هل هناك حد معين وضعته من الساعات لاستخدام الهواتف الذكية / الأجهزة اللوحية او مشاهدة التلفزيون؟

نعم

لا

9-ان كانت الإجابة نعم هل ابنك يتقبل هذه الحد بدون تمرد وعصيان؟

نعم

لا

10-متى تسمح لطفلك باستخدام الأجهزة الاليكترونية او مشاهدة التلفزيون؟

في أي وقت يريده الطفل

عند انشغالي بعمل ما

عندما أكون مرهقة او في مزاج سيء

عند تقديم الطعام له

عندما يكون غاضب ويصرخ

عندما اريده ان ينام

أخرى

11-هل يستخدم أحد الهواتف الذكية / الأجهزة اللوحية او مشاهدة التليفزيون عند تناول وجباته؟

نعم

لا

12-هل يعاني ابنك من السمنة؟

نعم

لا

13-هل يمارس ابنك أي نشاط بدني رياضي؟

نعم

لا

إذا كانت الإجابة ب "نعم"

14-ممارسة النشاط البدني تكون بصفة

يومية

أسبوعيا

غير منتظم

15-ما هو نوع النشاط البدني الذي يمارسه طفلك؟

ركوب الدراجة

لعب الكرة

الجري

السباحة

الكاراتيه

أخرى

16-هل تستخدم الهواتف الذكية / الأجهزة اللوحية كنوع من التحفيز لابنك إذا عمل شيء صحيح؟

نعم

لا

أحيانا

17-هل تمنع طفلك من استخدام الهواتف الذكية / الأجهزة اللوحية إذا أخطأ في شيء؟

نعم

لا

احيانا

18-هل يستخدم طفلك الهواتف الذكية / الأجهزة اللوحية او مشاهدة التلفزيون من قبل النوم؟

نعم

لا

احيانا

19-هل ينام ابنك قبل الساعة التاسعة مساء؟

نعم

لا

20-هل تحفز ابنك على مشاركة الأنشطة واللعب مع اقرانه بدلا من البقاء على اجهزتهم؟

نعم

لا

مواقف واتجاهات الإباء والامهات بشأن تنظيم تعرض أطفالهم تحت سن السادسة للشاشة في المنطقة الغربية، المملكة العربية السعودية

1- اقلق بشأن استخدام الهواتف الذكية / الأجهزة اللوحية من قبل أطفالك

اوافق

ربما

لا اوافق

2-ارى انه من الواجب عليك تحديد المحتوى الذي يشاهده ابنك

اوافق

ربما

لا أوافق

3-أجد صعوبة في التحكم في استخدام طفلك للهواتف الذكية / الأجهزة اللوحية او التليفزيون خاصة في وجود العديد من هذه الأجهزة في المنزل

أوافق

ربما

لا أوافق

4-أجد صعوبة في مراقبة استخدام طفلك للهواتف الذكية / الأجهزة اللوحية او التليفزيون نتيجة انشغالك بالعمل

أوافق

ربما

لا أوافق

5- انزعج من اكثار طفالك من تناول الاكل اثناء الانشغال باستخدام الهواتف الذكية / الأجهزة اللوحية او مشاهدة التليفزيون

أوافق

ربما

لا أوافق

6- اشعر بان وجود الهواتف الذكية / الأجهزة اللوحية في المنزل يقلل من الوقت الذي تقضيه الاسرة في التحاور او اللعب مع اطفالها

أوافق

ربما

لا اوافق

معرفة الإباء والامهات بشأن تنظيم تعرض أطفالهم تحت سن السادسة للشاشة في المنطقة الغربية، المملكة العربية السعودية

1-في أي عمر يمكن تعريض الأطفال للشاشات (الهواتف الذكية / الأجهزة اللوحية او التلفزيون)؟

تحت سن 18 شهرًا

من سن 18 شهرًا إلى 2 سنوات

من سن 2 إلى 5 سنوات

من سن 6 سنوات وما فوق

لا اعرف

2-كم يكون الوقت المسموح للطفل الذي يتم قضاؤه على الشاشة؟

غير محدود

ساعة واحدة في اليوم او اقل

ساعتان في اليوم

ثلاث ساعات فأكثر

3-ما المدة المسموح بها لمشاهدة التليفزيون او الأجهزة الاليكترونية قبل النوم؟

يمكن للطفل ان ينام وهو يشاهد التليفزيون او الأجهزة الاليكترونية الاخرى

لا تقل عن ساعة

لا تقل عن ساعتين

لا اعرف

4-هل استخدام الهواتف والأجهزة الاليكترونية بكثرة يؤدي الى ادمان استخدامها؟

نعم

لا

لا اعرف

5-هل استخدام الأطفال للهواتف الذكية / الأجهزة اللوحية يزيد من خطر الإصابة بالسمنة وأمراض أخرى كالسكري؟

نعم

لا

لا اعرف

6-هل استخدام الأطفال للهواتف الذكية / الأجهزة اللوحية يؤثر سلباً على صحتهم العقلية والجسدية؟

نعم

لا

لا اعرف

7-هل استخدام الهواتف الذكية / الأجهزة اللوحية يؤثر على جودة النوم لدى الطفل؟

نعم

لا

لا اعرف

8-هل استخدام الأطفال للهواتف الذكية / الأجهزة اللوحية يؤثر على تركيزهم وانتباههم؟

نعم

لا

لا اعرف

Questionnaire in English

Would you like to participate in the study while ensuring that we will not publish  any personal information about the participants? 

• Yes 

• No 

Demographic data 

1- Residence 

• Makkah and its governorates 

• Medina and its governorates 

• Outside the western region 

2-Age in Years 

3-Gender 

• Male 

• Female 

4-Nationality 

• Saudi 

• Non-Saudi 

5-Social status of parents 

• Husband 

• Wife 

• Separated 

• Widowed 

6-Academic qualifications 

• Primary School 

• Intermediate School 

• Secondary School 

• Diploma 

• University 

• Postgraduate 

7- Monthly income 

• Less than 5000 SAR 

• 5000-10000 SAR

• 10000-20000 SAR 

• More than 20000 SAR 

8-Profession 

• Government job 

• Housewife 

• Private job 

• Student 

• Independent business 

9-Do you have a child under the age of six years?

• Yes 

• No  

10- If you have, please mention gender of your children under age of six years.

• Male 

• Female 

11- How old is your child under six years? 

12- Does your child go to kindergarten? 

• Yes  

• No 

Parental practices regarding regulating screen exposure of under the six-year-old children in the Western Region, Kingdom of Saudi Arabia

1- Does your child have his/her own device? 

• Yes. 

• No. 

2- What type of devices used by your child under- age of six year?

• Smart phone. 

• iPad. 

• PlayStation/Xbox. 

• Computer/Laptop. 

3- Do you permit your child to watch TV/Smart Phones/Tablets?

• Yes. 

• No. 

4- How much time allowed by the child to spend on screen daily?

• Less than one hour. 

• 1-2 hours. 

• 2-3 hours. 

• More than 3 hours. 

5- Does device use by your child increased after the COVID-19 pandemic?

• Yes. 

• No. 

• May be. 

6- What is the content watched by your child under six years?

• Video games. 

• You tube. 

• Tik Tok. 

• Snapchat. 

• Instagram.

• Cartoon films. 

• Songs/chants for children. 

• Others. 

7-Do you allow son to use devices near you? 

• Yes. 

• No. 

• May be. 

8- Do you set a time limit for your child to use the device or watch TV?

• Yes. 

• No. 

9- If your answer is “Yes”; Does your child accept this limit without disobedience?

• Yes. 

• No. 

10- When do you allow your child to use electronic devices or watch TV?

• At any time, the child wants.

• When I am busy in my work.

• When I am tired or have bad mood.

• When feeding the child.

• When the child is angry or scream.

• When I want him to sleep.

• Others. 

11- Does your child watch screen while eating a meal?

• Yes.

• No.

12- Does your son suffer from obesity?

• Yes.

• No.

13- Does your son engage in physical activity?

• Yes.

• No.

If your answer” Yes”

14- How frequent does your child practice physical activities?

• Daily.

• Weekly.

• Irregular.

15- What type of physical exercise does your child do?

• Ridding bike.

• Playing ball.

• Running.

• Swimming.

• Karate.

• Others.

16- Do you motivate children with smartphones or tablets as a reward?

• Yes.

• No.

• Sometimes.

17- Do you forbid your child from using a device or watching TV before bedtime?

• Yes.

• No.

• Sometimes. 

18- Does your child use a device or TV just before bedtime?

• Yes.

• No.

• Sometimes.

19- Does your child sleep early at night (before 9 pm)?

• Yes.

• No.

20- Do you motivate your child to participate in physical activities and play with  his/her peers instead of staying on the devices?

• Yes.

• No.

Attitudes of parents regarding regulating the exposure of their children under  the age of six to the screen in the Western Province, Kingdom of Saudi Arabia

1- Do you worry about the use of smartphones/tablets by your children?

• Agree. 

• Neutral.

• Disagree.

2- Do you think it is your duty to limit the content that your son watches?

• Agree.

• Neutral.

• Disagree.

3- Do you find difficulty to control your child's use of smartphones/tablets or  television, especially when there are many of these devices at home?

• Agree.

• Neutral.

• Disagree.

4- Do you face difficulty to monitor your child’s use of smartphones/tablets or  television due to your busyness with work? 

• Agree.

• Neutral.

• Disagree.

5- Have you got annoyed by your child eating a lot while busy using  smartphones/tablets or watching TV?

• Agree.

• Neutral.

• Disagree.

6- Do you feel that the presence of smartphones/tablets at home reduces the  time the family spends communicating or playing with its children?

• Agree.

• Neutral.

• Disagree.

Parents’ knowledge about regulating screen exposure for their children under  the age of six in the Western Province, Saudi Arabia

1- At what age can children be exposed to screens (smartphones/tablets or TV)?

• Under the age of 18 months.

• Ages 18 months to 2 years.

• From 2 to 5 years old.

• Ages 6 years and above.

• I don't know.

2- How much time is a child allowed to spend on screen?

• Unlimited. 

• One hour a day or less.

• Two hours a day.

• Three hours or more.

3- How much time is allowed to watch television or electronic devices before sleeping?

• The child can sleep while watching television or other electronic devices.

• Not less than an hour. 

• Not less than two hours. 

• I don't know. 

4- Does excessive use of phones and electronic devices lead to addiction?

• Yes. 

• No. 

• I do not know. 

5- Does children's use of smartphones/tablets increase the risk of obesity and  other diseases such as diabetes? 

• Yes. 

• No. 

• I do not know. 

6- Does children's use of smartphones/tablets negatively affect their mental and  physical health? 

• Yes. 

• No. 

• I do not know. 

7- Does the use of smartphones/tablets affect a child's sleep quality?

• Yes. 

• No. 

• I do not know. 

8- Does children’s use of smartphones/tablets affect their concentration and  attention? 

• Yes. 

• No. 

• I do not know.

## References

[1] Ansari M (2019). WHO guidelines on physical activity, sedentary behaviour and sleep for children under 5 years of age. https://www.who.int/publications/i/item/9789241550536.

[2] (2023). Pew Research Center: Teens and Technology. Technology.

[3] Fuller C, Lehman E, Hicks S, Novick MB (2017). Bedtime use of technology and associated sleep problems in children. Glob Pediatr Health.

[4] Carson V, Hunter S, Kuzik N (2016). Systematic review of sedentary behaviour and health indicators in school-aged children and youth: An update. Appl Physiol Nutr Metab.

[5] Poitras VJ, Gray CE, Borghese MM (2016). Systematic review of the relationships between objectively measured physical activity and health indicators in school-aged children and youth. Appl Physiol Nutr Metab.

[6] Chaput JP, Gray CE, Poitras VJ (2016). Systematic review of the relationships between sleep duration and health indicators in school-aged children and youth. Appl Physiol Nutr Metab.

[7] Zhou M, Zhu W, Sun X, Huang L (2022). Internet addiction and child physical and mental health: Evidence from panel dataset in China. J Affect Disord.

[8] Engberg E, Figueiredo RA, Rounge TB, Weiderpass E, Viljakainen H (2019). Heavy screen users are the heaviest among 10,000 children. Sci Rep.

[9] Page AS, Cooper AR, Griew P, Jago R (2010). Children's screen viewing is related to psychological difficulties irrespective of physical activity. Pediatrics.

[10] Hutton JS, Dudley J, Horowitz-Kraus T, DeWitt T, Holland SK (2020). Associations between screen-based media use and brain white matter integrity in preschool-aged children. JAMA Pediatr.

[11] Guerrero MD, Barnes JD, Chaput JP, Tremblay MS (2019). Screen time and problem behaviors in children: Exploring the mediating role of sleep duration. Int J Behav Nutr Phys Act.

[12] Nwankwo F, Shin HD, Al-Habaibeh A, Massoud H (2019). Evaluation of children's screen viewing time and parental role in household context. Glob Pediatr Health.

[13] (2023). The American academy of child and adolescent psychiatry Updated February 2020 No. 54 Screen Time and Children. https://www.aacap.org/AACAP/Families_and_Youth/Facts_for_Families/FFF-Guide/Children-And-Watching-TV-054.aspx.

[14] (2023). The American Academy of Pediatrics (AAP) Center of Excellence on Social Media and Youth Mental Health. https://www.aap.org/en/patient-care/media-and-children/center-of-excellence-on-social-media-and-youth-mental-health/.

[15] Almaqhawi A, Albarqi M (2022). The effects of technology use on children's physical activity: A cross-sectional study in the Eastern province of Saudi Arabia. J Med Life.

[16] Dessie M, Techane MA, Tesfaye B, Gebeyehu DA (2021). Elementary school teachers knowledge and attitude towards attention deficit-hyperactivity disorder in Gondar, Ethiopia: A multi-institutional study. Child Adolesc Psychiatry Ment Health.

[17] Schoppe-Sullivan SJ, Kotila L, Jia R, Lang SN, Bower DJ (2013). Comparisons of levels and predictors of mothers' and fathers' engagement with their preschool aged children. Early Child Dev Care.

[18] Ghorbani S, Gharraee B, Hosseini F, Maghami Sharif Z, Aghebati A (2022). Changing parenting style between two generations and its impacts on the severity of behavioral and emotional symptoms. Asia Pac Psychiatry.

[19] Eales L, Gillespie S, Alstat RA, Ferguson GM, Carlson SM (2021). Children's screen and problematic media use in the United States before and during the COVID-19 pandemic. Child Dev.

[20] Kamaleddine AN, Antar HA, Ali BT (2022). Effect of screen time on physical and mental health and eating habits during COVID-19 lockdown in Lebanon. Psychiatry Investig.

[21] Serra G, Lo Scalzo L, Giuffrè M, Ferrara P, Corsello G (2021). Smartphone use and addiction during the coronavirus disease 2019 (COVID-19) pandemic: cohort study on 184 Italian children and adolescents. Ital J Pediatr.

[22] Alqarni TA, Alshamrani MA, Alzahrani AS, AlRefaie AM, Balkhair OH, Alsaegh SZ (2022). Prevalence of screen time use and its relationship with obesity, sleep quality, and parental knowledge of related guidelines: A study on children and adolescents attending Primary Healthcare Centers in the Makkah Region. J Family Community Med.

[23] Carson V, Janssen I (2012). Associations between factors within the home setting and screen time among children aged 0-5 years: A cross-sectional study. BMC Public Health.

[24] Madigan S, Browne D, Racine N, Mori C, Tough S (2019). Association between screen time and children's performance on a developmental screening test. JAMA Pediatr.

[25] Sauce B, Liebherr M, Judd N, Klingberg T (2022). The impact of digital media on children's intelligence while controlling for genetic differences in cognition and socioeconomic background. Sci Rep.

[26] Nathan T, Muthupalaniappen L, Muhammad NA (2022). Prevalence and description of digital device use among preschool children: A cross-sectional study in Kota Setar District, Kedah. Malays Fam Physician.

[27] Radesky JS, Peacock-Chambers E, Zuckerman B, Silverstein M (2016). Use of mobile technology to calm upset children: Associations with social-emotional development. JAMA Pediatr.

[28] Rakha AH, Albahadel DM, Saleh HA (2022). Developing an active lifestyle for children considering the Saudi vision 2030: The family's point of view. PLoS One.

[29] Vereecken CA, Keukelier E, Maes L (2004). Influence of mother's educational level on food parenting practices and food habits of young children. Appetite.

[30] Mohamed N, Soliman S, Abd El-Mouty S (2021). Mothers' knowledge and practice regarding electronic media used by their children. Mansoura Nurs J.

[31] Goncalves WS, Byrne R, Viana MT, Trost SG (2019). Parental influences on screen time and weight status among preschool children from Brazil: A cross-sectional study. Int J Behav Nutr Phys Act.

